# The Importance of Infant Feeding

**Published:** 1906-07

**Authors:** 


THE IMPORTANCE OF INFANT FEEDING.—With the oncoming of the warm season will come the intestinal disorders of infants, which are the cause of such a high mortality rate amongst the young. The breast-fed baby, as a rule, finds adequate sustenance, but to the infant who has to rely on artificial feeding life is a struggle from the start. Unfortunately, practitioners neglect to give this very important problem the study it deserves, and so when the anxious mother seeks advice she is too often told to try one or the other of the various advertised proprietary foods, and to follow the directions on the label. The management of the baby's food is no simple matter, and each case must be carefully studied, and often it is only after long and arduous study that the proper adjustment of fats, proteids and carbohydrates can be found. It is vastly more difficult to prescribe food for an infant than it is to prescribe drugs. Of the few gratifications that come to the physician, none is greater than that of developing a poor, emaciated infant into a fat and happy baby merely by the proper management of its food supply.—*Medical Age.*

